# Public health nurses experience of mental health encounters in the context of primary health care: a constructivist grounded theory study

**DOI:** 10.1186/s12912-023-01340-7

**Published:** 2023-05-25

**Authors:** Emmy Nilsson, Suzanne Johanson, Lina Behm, Ulrika Bejerholm

**Affiliations:** 1 Department of Health Sciences/Mental Health, Activity and Participation, Lund University, Lund, Sweden; 2 Department of Nursing and Integrated Health Sciences, Kristianstad University, Kristianstad, Sweden; 3 Department of Research and development, Region Skåne, Mental Health Services, County Council of Skåne, Malmö, Sweden

**Keywords:** Clinical nursing research, Public health nurse, Mental health literacy, Mental health services, Primary health care, Health promotion

## Abstract

**Background:**

In primary health care people with mental health needs are often overlooked or masked with physical complaints. It has been suggested that public health nurses lack sufficient knowledge when encountering people with mental health problems. Low levels of mental health literacy among professionals are associated with negative patient outcome. There is a need to understand public health nurses process and strategies used when encountering a person with mental health problems in order to promote mental health. This study aimed to construct a theory that explains the process of public health nurses experience when encountering people with mental health problems based on their knowledge, attitudes, and beliefs about mental health.

**Methods:**

A constructivist grounded theory design was used to meet the aim of the study. Interviews were conducted with 13 public health nurses working in primary health care between October 2019 and June 2021, and the data analysis was performed according to the principles of Charmaz.

**Results:**

The core category, *“Public health nurses as a relationship builder – to initiate the dialogue”* reflected the process while the main categories “Being on your own*”*, *“Being on top of things- knowing your limits”*, and “Professional comfort zone*”* reflected conditions that were decisive for initiating a dialogue.

**Conclusion:**

Managing mental health encounters in primary health care was a personal and complex decision-making process that depends on the public health nurses’ professional comfort zone and acquired mental health literacy. Narratives of the public health nurses helped to construct a theory and understand the conditions for recognizing, managing and promoting mental health in primary health care.

**Supplementary Information:**

The online version contains supplementary material available at 10.1186/s12912-023-01340-7.

## Background

The rationale of this study is to explore the knowledge, attitudes, and beliefs about mental health (MH) of public health nurses (PHN) when encountering peoples with mental health problems (MHPs) and how the PHNs deal with the situation in the context of primary health care (PHC). We use the World Health Organization’s definition: “*mental health is a state of well-being in which an individual realizes his or her own abilities, can cope with the normal stresses of life, can work productively and fruitfully, and is able to make a contribution to his or her community”* [1]. MHP is a broad concept encompassing many aspects from challenges experienced in everyday life to severe mental disorders [1]. People with MHPs are often overlooked in PHC [2]. Their mental health needs are masked with physical complaints and co-occur with other disorders that are more manifest [3–5]. Furthermore, the decision support system does not automatically recognize MHPs [6] and there are few interventions that exist that promote mental health [7]. It has been suggested in a number of studies that PHNs lack sufficient knowledge about MHPs and about how they encounter MH patients and promote MH [6, 8–10]. Moreover, a low level of mental health literacy (MHL) among professionals is associated with negative patient outcomes [11, 12]. Mental health literacy is defined here as knowledge, attitudes and beliefs about MHPs, and strategies used i.e. recognition, management and prevention of MHPs [12]. MHL is used to promote PHNs’ knowledge and their ability to benefit the MH perspective of the patients they encounter. It has been emphasized that being a capable and useful nurse are important characteristics when encountering persons seeking care for their MHPs [6]. There is a need to understand the knowledge, attitudes, and beliefs about MH of PHNs in their encounters with persons with MHPs in a PHC context.

There is an increase of citizens with MHPs in Sweden [13, 14] and there is a risk of a further increase in MHPs due to the Covid-19 pandemic [15]. MHPs are known to be associated with higher levels of smoking, more frequent alcohol consumption, and a greater degree of obesity [16] which can further cause deterioration in a person’s overall health [17, 18]. Persons with MHPs also have a higher risk of illicit substance use [19]. Furthermore, people with MHPs, have a greater risk of suicide than the general population [20, 21]. At the same time, encounters between staff and patients has been identified as being the most frequently occurring deficiency in PHC and MH services prior to a patient’s suicide, according to Roos af Hejlmsäter et al. [22]. PHC as the first-line of MH services have the opportunity to play an important role [23] in providing early support and prevention to promote health to a group of patients who are known to be in a vulnerable position in society today [1, 24].

The responsibility of providing MH care is evenly shared between the first-line PHC service and the specialist MH services [25]. The National Board of Health and Welfare emphasizes the importance of defining MH needs and providing MH promotion and preventive activities in a PHC context [23]. Common barriers for integrating MH into a PHC context have shown to be attitudes, knowledge and skills, motivation to change, management and leadership, and resources [26]. To access MH services in this context is perceived as a challenge for patients and similarly difficult for the providers across PHC services to maintain it [26, 27]. The nursing role in PHC is a diverse one and the spheres of responsibility vary across organizations, counties, and countries [28, 29]. In addition, how nurses perform their work can vary from one to another, and the essential procedures for working with patients with MHP within the PHC have not been sufficiently evolved [2].

The PHNs’ primary focus is health promotion and prevention from a public health perspective [30, 31]. They describe their function in PHC centers as being the glue that holds the health care service together for patients with MHPs seeking support [9]. However, PHNs lacked confidence [32] and felt insecure about how to meet the MH needs of the patients, and were uncertain of their role [9]. More focus is needed on mental health promotion according to the Swedish Association of Local Authorities and Regions [33]. Mental health promotion is not currently seen as a natural part of the care provided by the PHC for the population [8, 15, 34].

There is thus a need to understand the knowledge, attitude and views of PHNs, as front-line workers within PHC, from identifying to assessing a person’s MH [15, 35] to meeting the challenges PHNs face as the MH needs are being integrated within PHC. The aim is to construct a theory that explains the process public health nurses experience when encountering people with MHPs, based on their knowledge, attitudes, and beliefs about mental health.

## Methods

### Study setting

A constructivist grounded theory study design [36] was used due to the limited existing knowledge and prior inductive theory of PHNs’ experience of encounters with persons with MHPs. Grounded theory is relevant when a theory is needed to explain actions and processes of a specific situation [36]. The process and the actions taken by PHN in a PHC context can have multiple perspectives, and we therefore choose a constructivist approach where the researchers are part of the construction and aware of the changing context and different perspectives of reality [36, 37]. The consolidated criteria for reporting qualitative research (COREQ) were used [38]. The selection of PHC settings were based on the PHNs’ working field defined by The Swedish Society of Nursing [30]. The PHC centers were situated in small and medium sized cities while the municipal school health care settings were situated in larger cities.

### Participants and sampling

According to the standards of grounded theory [36], the participants, PHNs, were gradually included in the study, initially using purposeful sampling and later a focused theoretical sampling procedure. The intention was to approach PHNs working in different PHC contexts with varied experience of the field. The theoretical sampling process involved presumptive participants of different ages from different PHC contexts, with varying work experience as PHN and experience of encounters with people with MHPs. During the theoretical sampling process, we were also interested in interviewing PHN students working in PHC, with specific experience of encounters with people with MHPs in order to understand the knowledge development process of MH.

### Data collection

The data collection was performed from October 2019 to June 2021. A total of 13 interviews with 13 participants were completed. The interviews, which were performed by the first (E.N.) and third author (L.B.), who are also PHNs, were conducted face-to-face in accordance with the choice of the participants. The location for the interviews changed to an online meeting service at Lund University, LU-Zoom, communicated via a LU IP address when the Covid-19 pandemic emerged and reduced the possibilities for physical meetings. The data was encrypted and all users need a specific password in order to log in. The interviews lasted approximately 56 min (range 37–67 min) and were digitally recorded after consent from each participant. The interview guide was constructed by the authors and were based on the MHL concept with a focus on PHNs primary, secondary, and tertiary health promotion and preventive working field see Table 1. The first author (E.N.) contacted operational managers in the PHC services to inform and invite them to participate in the study. The PHC service that accepted the invitation then contacted PHNs and the first (EN) and second author (LB) then approached those who showed a positive interest in participating in the study. Minor revisions of the interview guide took place after two pilot interviews were conducted. In the minor revisions, questions of organizational structure and support for the PHN were added. All the participants had received information about the study in advance and were also orally introduced prior to the interview being conducted with a short background of the study’s aim and that the study was a part of a larger project. A brief and standardized background narrative was provided in order to introduce the participants to the field of interest. The early stages in the analysis were performed by all authors during the interview period. The analysis included initial coding, memo writing, and the development of preliminary concepts [36]. This analytical process resulted in modifications to the questions in the interview guide that corresponded to the topics raised during the interviews. The initial stage of the analysis process further guided the theoretical sampling.

**Table 1:** Examples of questions and probing questions in the interview guide

Primary prevention
How would you identify that the person is seeking help for mental health problems?
Could you describe the last encounter you had with a patient with MHPs?
Secondary prevention
Could you describe how you identify risk factors for MHPs?
Can you describe how you work with mental health promotion and prevention at your workplace?
Tertial prevention
Could you describe the care you as a RN/PHN offer patients with MHP?
Could you describe which support you as a PHN have from your employer to support patients with MHPs?
Probing questions
Could you tell me more?
When we were discussing … What was your impression?
You mentioned XXX, can you please explain what you mean by that?

### Data analysis

The coding process was based on theoretical sampling, coding, constant comparison, identification and data saturation [36, 37]. The memos written during the interviews were used to go back and forth in the data to construct meaning and actions, as an early analysis of the data. The memos were sorted to analyze their relationship and relative significance to each other i.e., clustering. Theoretical sampling was used to develop and respond to new question topics that had evolved from the initial coding and analyses. All authors contributed to this early step of the analytical process. In order for the results of the process and the actions to emerge from the data, codes were analyzed to construct the data into forms of concepts, see Table 2. Categorization was made by constant comparisons between the codes and concepts that referred to the process of encountering people with MHP. The interviews were transcribed verbatim, and line-by-line coding was conducted. NVivo 12. 2 software was used as a tool to organize data, as well as to store it. The initial coding was performed by the first author (E.N.), while two authors (S.J., L.B.) replicated the coding of four randomly chosen interview documents separately. This was carried out to validate the initial coding and consensus was found during a meeting. The last author (U.B.) then analyzed the emerging concepts and the present theory based on an initial analysis of the coding. A workshop was then convened where the authors further explored the material and established relationships between the coding. Meaning was revealed by exploring patterns between codes. A definition of the properties and dimensions of the tentative main categories were constructed by asking how they were related. The core category and main categories were constructed by all the authors who were active in the development of the process of understanding how PHNs’ attitudes, views, beliefs, and knowledge was used in the encounters with people with MHPs.

**Table 2:** Example of the analysis process

Text from the Interview	Initial coding	Properties /Tentative coding	Concepts	Subcategory	Main category	Core category
“think it is all about who you are. What kind of personal history you bring into the encounter. If you’re used to talking about emotions and feelings in your personal life, you’ re more comfortable talking about in your professional role”. (10)	Who you are as a person Personal history you bring	• Depends on who you are as a person • Personal and professional • Experience matter	• Desirable attributes Previous experience	Desirable attributes Previous experience- learning by doing	Being on top of things – knowing your limits	Being a relationship builder – to initiate the dialogue
“Yes, if you look at our guidelines it consists of a lot of physical health, measurements of some sorts, but very little of MH, how to approach MH encounters. I think it’s because MH is a lot more difficult to capture. It is easier to do guidelines based on measurements, like physical health”. (9)	MH is difficult to capture Very little of MH	• Lack of organizational support Physical vs Mental health positions in the organization	Knowledge gap	• Mental health does not fit—Organizational unreadiness	Being on your own – A knowledge gap	
**Respondent:** “Yes. When it comes to MH, we’re more like this needs to be taken care of by someone else, we have a responsibility to connect the patient to the counselor or the physician”.** (**3)	Needs to be taken care of by someone else	Professional boundaries	To guide and refer to the right person	The intermediator	Professional comfort zone	
	We have a responsibility to connect					

### Trustworthiness

The guidelines for enhancing trustworthiness and quality were used to enhance the study’s trustworthiness [38, 39]. The criteria for evaluation are credibility, originality, resonance, and usefulness [36]. The criteria of credibility were strengthened by using a theoretical sampling over a period of one year, interviewing a variety of PHN within different fields and with a range of experience as a PHN or in the process of becoming a PHN. We had a good variety of range and interview depth in the data and all the authors contributed to the construction of the social process. To meet the criteria of originality, our result has provided new insights into a very limited area of knowledge of PHNs dealing with patients with MHPs. In the reinterviews the participants’ confirmed the presented theory and had no additional comments, therefore no amendments were made. To meet the criteria of usefulness, the presented theory helps to illuminate PHNs’ encounters with persons with MHPs which knowledge can contribute to stakeholders’ and health professionals’ assimilation of useful knowledge of how to promote MH in PHC and create meaningful encounters with persons with MHPs.

## Results

The participants’ ages ranged from 30- 60 years (mean 41 years) (see Table 3), with a range of working experience as a PHN of < 0—36 years (mean 11 years). The 13 interviews and three reinterviews after the final drafts of the result, resulted in the core category, *“PHN as a relationship builder- to initiate the dialogue”* and was constructed by three main categories; *“*Being on your own*”, “Being on top of things –knowing your limits*” and *“*Professional comfort zone*”.* The main categories had two or three subcategories and are presented in turn and exemplified by quotes from the participants.

**Table 3:** Socio-demographic characteristics of the participants

	**Age**^**a**^	**Educational background RN = Registered nurse, PHN = Public health nurse**^**a**^	**Experience as Registered Nurse**^**a**^	**Years worked at current PHC **^**a**^	**Experience as PHN**^**a**^
1	43	RN, PHN	> 10	5–7	2
2	42	RN, PHN	3–5	3–5	1
3	35	RN, PHN student	> 10	3–5	0
4	61	RN, PHN and ^b^	> 10	3–5	36
5	61	RN, PHN	> 10	5–7	35
6	30	RN, PHN	5–7	5–7	2
7	45	RN, PHN	> 10	7–9	15
8	59	RN, PHN and ^b^	> 10	5–7	20
9	42	RN, PHN	> 10	3–5	1
10	40	RN, PHN	> 10	1–3	< 1
11	44	RN, PHN	> 10	3–5	6
12	49	RN, PHN	> 10	3–5	12
13	56	RN, PHN	> 10	0–1	18
Mean (range)	47 (30–61)		> 10	3–5	11 (0–36)

^**a**^**Years**

^**b**^**Additional Specialist training**

### The core category PHN as a relationship builder – to initiate the dialogue

**Fig. 1:**
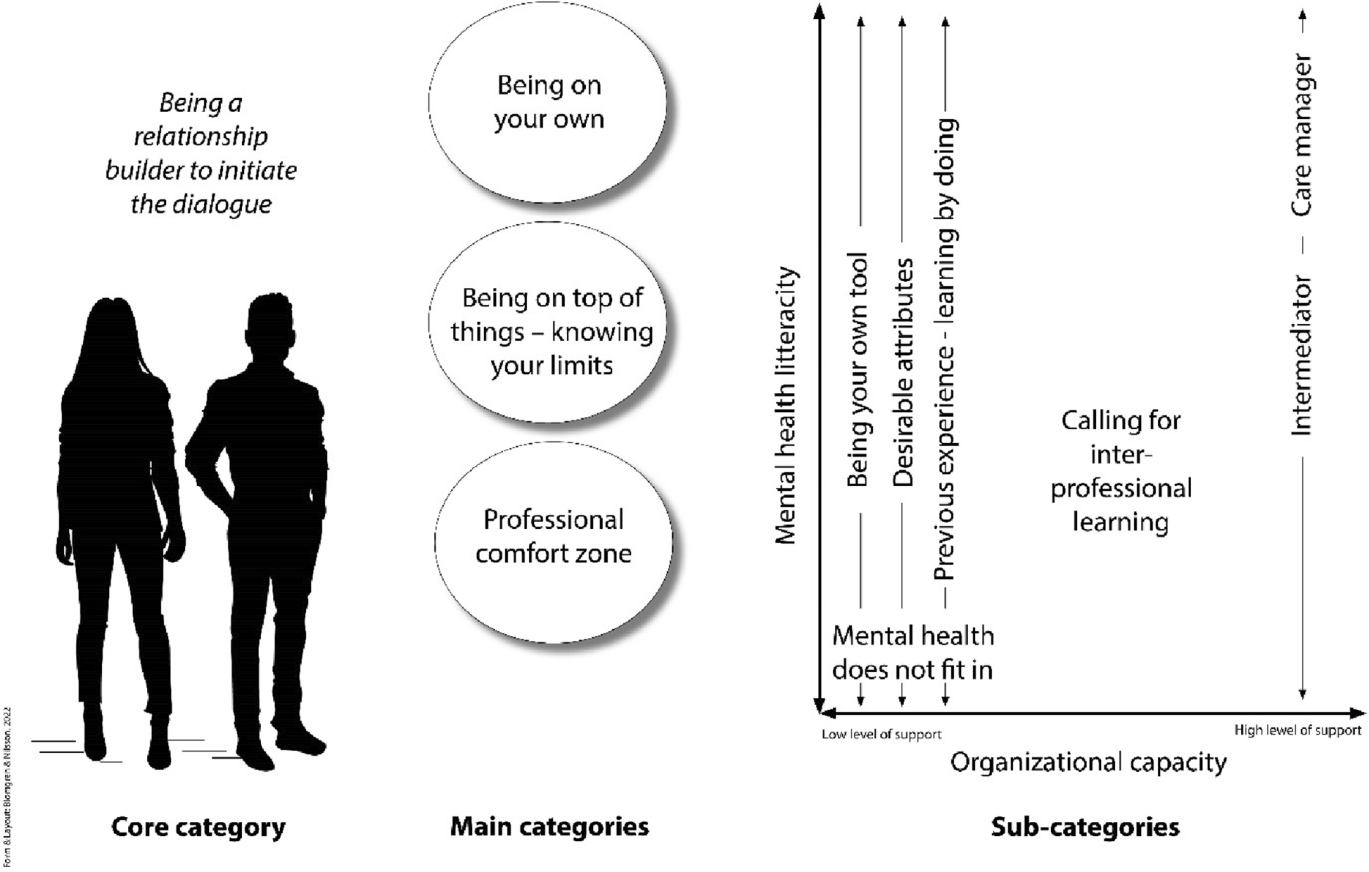
The process of being a relationship builder to initiate a dialogue should be viewed in the light of the organizational capacity with low to high support for the encounter and the PHNs’ MHL. The subcategories merge into two main categories, and together they construct the third main category, the PHN professional comfort zone where different actions take place depending on PHNs’ MHL and the organizational capacity

Being a relationship builder- to initiate the dialogue concerned the effort of creating a caring relationship, was key to recognizing, guiding, or referring and managing patients with MHP. However, the prerequisites for being able to create caring relationships differed due to the inter-related main categories of *“*Being on your own” and “*Being on top of things – knowing your limits*”, which emerged to a third main category, “Professional comfort zone*”,* see Fig. 1.

The PHNs described an existing knowledge gap concerning MH in the organization and here was a knowledge diversity among PHNs. There was a different structure of dealing with MH within a PHC organization and between PHC organizations, which created an uncertainty of PHNs’ professional role. To close the knowledge and MH service gap, the PHNs claimed the need for a change of the organizational structure, resources, and management support. Desirable attributes were reflected among those PHNs who had dealt with and managed encounters concerning MH more willingly than others, a social construction that was a result of the lack of organizational capacity, were mental health did not fit in*.* Hence, the PHNs’ professional role felt uncertain, and they were caught between the patient and the organization, which are, described in the main categories of *“Being on top of things- knowing your limits”* and “Being your own tool*”*. The main category “Professional comfort zone*”* reflected how the PHNs dealt with MH encounters, and how to bridge the knowledge gap of MH.

**Fig. 2:**
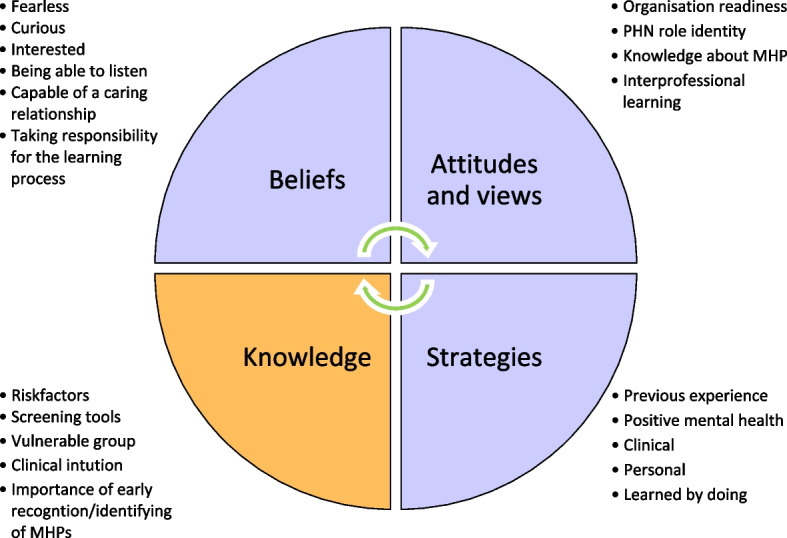
Factors of mental health literacy described by public health nurses in encounters with patients with mental health problems

Figure 1 represents PHNs’ MHL (vertical) and the organizational capacity (horizontal). The operationalization reflects different approaches and actions of the PHN in the encounter, described as *the Intermediator* and *the Care Manager*, and is a result of the PHN acquiring MHL. Higher levels of MHL and available capacity in the organization were related to greater chances of taking actions as a care manager and vice versa. When PHNs were identified as having lower levels of MHL and the organizational capacity was lacking, the PHN became an intermediator. It was easier to initiate a dialogue when the organization supported such actions. Furthermore, it was also considered easier to work as *a care manager* if there was a balance between *“*Being on your own*”* and *“Being on top of things- knowing your limits”.* Not all PHNs had the personal characteristics to deal with encounters with MHP, i.e., the *desirable attributes*, and tended to choose *the intermediator* role. To fully grasp the complex construction of being a relationship builder, different factors contributing to PHNs’ MHL (strategies and actions) are presented in Fig. 2.

### Being on your own

The main category of *“*Being on your own*”* describes how the PHNs dealt with the encounter as a relationship builder in relation to the organizational capacity of MH. The organizational capacity was weak and lacked structure, resources, support, and working culture, leaving the PHN on their own to enable a dialogue about MH. This is reflected by the two subcategories *Mental health does not fit in* and *Desirable attributes*.

### Mental health does not fit in

The PHC context reflected a two linear process of care; one from a physical health point of view, which was familiar to the PHNs, and a second from a MH perspective. The physical health care needs had, in comparison with the MH care needs, a given structure, resources, an adequate working environment, and formal written guidelines for the PHN to use during an encounter. Regarding MH, such resources were missing, i.e., lack of organizational capacity and thus opportunities for enabling a dialogue about MH. This led to an ethical struggle for the PHN who then prioritized and managed physical health care needs. MH care and encounters were time consuming and shifting the focus and time from physical health care needs was not always possible. The organization had not realized that increased resources and knowledge were needed to support MH. This lack of resources may have functioned as an excuse for not advocating or dealing with MHP at the PHC.

*“But when it comes to mental health, it is not at all that easy, you cannot do the same for all persons, there is no… what do you say… such a strict guidance as there is when it comes to physical health”* (Participant 9)

When the PHN had the option to make an active decision to provide care, little room (resources) was left for them to build a caring relationship with patients who had complex MH needs. The situation led to frustration among the PHNs that encounters were being made without mandate, support, knowledge, and timely strategies of how to deal with the situation.

*“Then it’s been difficult to find a time for the patient to be assessed (to see another professional), and then it may be that someone calls, and you can hear that they’re feeling bad and then there’s no time available for four weeks. Then it feels like it is a long time, but I have no power to… Yes, I could have booked the patient for myself, but I do not feel that I could have done so much more than just listening. So therefore…yes, you feel powerless sometimes when you can’t help them sooner”.* (Participant 7)

### Desirable attributes

*Desirable attributes* were a social construction for dealing with MH. PHNs were concerned and being curious, fearless, and open. The most crucial attribute was when they became particularly interested and invested in a patient. Under such circumstances, they took a responsibility for their own professional learning process and professional role development. Being fearless and having the ability to dare to take on conversations and to ask difficult questions were also critical for initiating a dialogue. The attributes described were also used to provide a positive description of colleagues who were more likely to take on encounters with MH. These were based on a description of a voluntary approach and not all PHNs had the *desirable attributes* when it came to dealing with MH encounters and this was considered as the right thing to do. Someone in the PHN group always had a special interest in MH.

*“We have a new girl here who’s only been a nurse for a year, she was a bit cautious, but now you just notice because she works here, that she like… she ends up in situations where she has to dare to ask questions. She has to be a little tough and she’s kind of just blossomed, and she asks such questions and deals with patients in such a fantastic way that I almost get a little teary-eyed when I think about it, because she’s so damn good.”* (Participant 2)

The importance of having *Desirable attributes* in an organization where *mental health does not fit in* where therefore emphasized. Being without such attributes and having little confidence and knowledge, created frustration, especially since health promotion and prevention are viewed as professional responsibilities that should be provided for their patients’ with MHPs.

### Being on top of things—knowing your limits

The PHNs’ ability to manage the encounter with patients with MHP was also characterized as *“Being on top of things – knowing your limits”,* which relates to how the PHNs described their knowledge of dealing with MHPs. Their knowledge about MH was related to *Being your own tool,* which was a description of clinical intuition where previous experience of dealing with encounters helped them learning by doing.

### Being your own tool

*Being your own tool* reflect how PHNs use themselves as a tool in terms of using open questions, characterized by motivational interview techniques, in their effort to maintain a non-judgmental attitude. Clinical intuition was one way of describing how to assess the patient in the encounter when they used themselves as a tool. This was described as something that could not be taught, rather as something they had gained through clinical experience, and this was an individual competence. Clinical intuition was critical for recognizing and identifying MHPs and enabled the PHN to understand the underlying meaning of sentences that were not always spoken verbally. The encounter could lead to several paths and solutions, which was in line with the understanding of nursing principles. The sense of security was something that grew with the number of years of clinical experience and was less concerned with previous or ongoing educational training. The answers of how to promote MH were not always present, but they knew how to listen, when to guide and refer patients to other team members or professionals in specialist care.

*“Something that I’ve realized in recent years is that you use… when you meet a person who’s ill or persons in general, you use so many senses, you use your eyes, smells and hearing. I usually describe it as having a lot of tentacles, where you sort of scan the person, and it’s a habit you get when you’ve worked with persons for many years.”* (Participant 8)

The quality of the encounter was greatly dependent on how PHNs were able to cope with their own life situation at that specific moment in time. To be able to listen, learn about and assess the person’s MHPs, the PHNs needed to pay careful attention. If the PHNs were *“*Being on top of things - knowing your limits*”,* they had the energy and dared to open up and initiate a dialogue about MH. A dialogue about MH took more energy from themselves than encounters of physical health.

### Previous experience – learning by doing

A majority of the experiences needed to promote MH concerned a learning by doing—approach. Lacking experience increased the uncertainty of the PHNs’ caring responsibility and was associated with a fear of doing wrong. Furthermore, the organizational capacity of providing PHC for persons with MHP, and the lack of MH training in the PHN education, made it difficult for them to manage the encounters. The clinical reasoning process during the encounter concerning decisions about which actions to take was related to and depending on if, the PHNs felt as *“Being on top of things – to know your limits”.* They could take active decisions to not become involved since the encounter was outside their *“*Professional comfort zone*”* when they felt they were not on top of things. The PHNs had developed their own way of dealing with the encounter in relation to the feeling or awareness of if they were on top of things.

*It is in our nature to want to fix things, but… here it is probably not to fix their well-being, it is to help them come right, so that they can feel better. Because I do not think that, I do not feel that I have the competence so that I can fix them.* (Participant 6)

Furthermore, personal experience could concern both the PHNs’ own and/or significant others’ experience of suffering from MHP. Personal experience helped the PHNs to understand the health care system from another point of view, and how difficult it can be to receive the “right kind of care”. Most of all, to reach out to the “right person” who could orchestrate the care. Having personal experience also contributed to the PHNs’ attitude towards the group with MHPs in general, whomever they expressed as being a vulnerable group who often appeared to fall between the cracks of the health care system.

*“Yes, I’ve got knowledge from different courses, which haven’t been very useful. The clinical training we had, has not been of much use. The knowledge I have is based on my own experiences, my own experience of MHPs, and experiences based on family members around me who have been ill or/and are ill.”* (Participant 11)

### Professional comfort zone

In the end, the encounter depended on the PHNs’ *“*professional comfort zone*”* which resulted into different actions depending on the organizational capacity and the PHNs’ MHL. *Calling for inter-professional learning* was described as a way forward for the PHN to take an active part in health promotion activities for all patients at the PHC and to be recognized by the PHC as experts in public health promotion and prevention activities. This category constitutes of three subcategories *The Care manager, The Intermediator* and *Calling for inter-professional learning***.**

### The intermediator

The PHNs role as an intermediator can be described as a spider in the web, as a coordinator of the care around patients with MHP. Referring patients was viewed as an act of care where the PHNs made sure that the patient reached the right person or level of care, directed by the organizational structure and the available resources. The clinical intuition guided them to refer the patient to another profession when the encounter was perceived to be outside of the *“*professional comfort zone*”.* Referring patients in these circumstances was viewed as the best option for the patient to move forward. PHNs could refrain from their own nursing responsibility with consideration for the well-being of the patient, while they at the same time limited their involvement. *The intermediator* role was also sometimes constructed and generated by the organization, as a function that should guide and refer individuals with MHP to someone else, either outside or within the organization depending on the organizational capacity.

*“I think it's difficult, it's very difficult, because you want to go into it… sometimes you can feel that they almost don’t want to tell me too much, because they know that I’m an intermediator. Therefore, it feels as though they will only talk properly and open up to the person who is really going to help them, so to speak. So therefore, I can feel that it is difficult to ask too many questions, because then you go too deep, and I can… I do not feel that I can help them with any advice or so, so it is so difficult…* (Participant 7*)*

### The care manager

Being a care manager meant that the PHNs took responsibility for the care for the patient with MHP and were dependent on organizational support in contrast to being an intermediator. As a care manager the PHN had resources that were available for taking actions based on their assessment, follow-ups etc. It became possible to create a caring relationship based on continuous care, and to build upon a mutual trust between the patient and the PHN. A care manager had, like *the intermediator*, a responsibility to refer patients to other professions but remained in support of the patients if they needed it. A care manager was someone with a mandate to make their own decisions (autonomy) and who was reliant on his/her clinical intuition and felt confident i.e., a high level of MHL in a role that was within their *“*professional comfort zone*”.* In such cases, the organizational capacity generated a flexibility for the PHN when the organizational structure and resources were in place. This flexibility made it possible for the PHNs to attempt using more unconventional actions to meet the patient needs during the encounter.

*“Mm. So that then I found an electronic advice to help you as a patient to take the right amount of your medication and at the right time, this one was locked, so the patient couldn’t open it, because that was it, the problem was the patient’s impulsiveness, and then the patient got the medication when he needed it and so we filled it once every four days, I think, something like that”.* (Participant 2)

On the other hand, being a care manager when the organizational capacity was short on resources, left the PHN to take actions that were outside of their *“*professional comfort zone”. Being forced to do this was felt as a betrayal against the patient’s trust. The PHNs could sometimes take actions as a silent protest against the organization’s inadequate capacity for MH. The silent protest was a desire to improve the situation for the patient but also to live up to the standards of being a PHN, to take time to listen and follow through*.* Their view of their professional role in the encounters with MHPs and the standard of being a PHN was connected to how the PHN viewed himself or herself as a person.

*“I don’t finish off a conversation because I know that there’s a red light on the phone, I don’t and I think so yes, I know that many… there are some who are affected by it and hurry up, but… I'm not that kind of person, so then… it's probably my little protest at the system”.* (Participant 1)

### Calling for inter-professional learning

The PHNs emphasized that one way of gaining knowledge was to be allowed to follow up on patient contacts, however, not being a natural part of the patient’s care process contributed to feelings of uncertainty. The importance of team building and collaborating with other professions that could contribute to PHN’s learning about managing patients with MHPs was also something the PHNs reflected on. However, the lack of collaboration within the PHC or between other health and care organizations concerning this group led the PHN to assume that this was not really their patient group, even though they emphasized the awareness that patients with MHP constitute a considerable proportion of the population in society today. Nonetheless, MH was part of the PHNs’ care agenda in terms of the specific target groups they were to work with, e.g., the elderly and children and/or parents at child health centers. Assessing the severity of MHP for these groups was part of the job. The PHNs considered themselves to be a temporary contact where the other encounters that were not part of their usual care work were concerned. Moreover, the PHN expressed a deep concern about how care was delivered in those encounters and a frustration for not being able to provide a standardized care.

*“Yes, it’s a group who have really been forgotten. It is a group who have been mistreated, I think; they do not get the right care. Not the help that they need and it's like there are many who fall between the cracks a little and it’s not really… it’s both healthcare centers and hospitals, it’s like… I do not know, but it feels a bit as though… It doesn’t matter so much with them, so it… yes, they aren’t taken care of in the way that you would like it to be, I think.”* (Participant 11)

Efforts had been made to improve knowledge about MHPs at their workplaces as well as to find different ways forward to improve the awareness and the care processes. However, certain factors prevented these efforts from being successful. Importantly, the manager needed to be interested, engaged and knowledgeable (*desirable attributes*), to embrace the complexity of MHP and the resources necessary.

*“So, we have become better and more accepting, it is like… the knowledge has increased, it’s more common now, but we don’t have the resources to monitor them. We find them more easily, but what do we do with them then? … Then they have to wait calmly and quietly for their turn.”* (Participant 10)

*Calling for inter-professional learning* was a description of how to improve knowledge about MH but also to incorporate the PHN in the care for patients with MHPs.

*“I imagine, my belief, my deepest belief is that I can hold the possible mental illness of a patient at a distance, if I can contribute with what I can, and try to provide a supportive conversation, mediate social contacts. So, then I think that it’s my job, to maybe make sure that X never gets there.”* (Participant 5)

## Discussion

This study explains the process of how PHNs in PHC encounter persons in the context of MHP. Their knowledge, attitude, and view on MH (mental health literacy) depended on whether the organization had the capacity for the PHN to build a relationship with the patient to initiate a dialogue about mental health, as reflected by the core category. In order to operationalize this, the decision-making process involved different steps based on the PHN’ MHL. Our understanding of the PHNs’ mental health literacy, as defined by Jorm [12], was that their knowledge of MH was closely linked to their own personal and/or clinical experience and personal characteristics of managing encounters with persons with MHPs. Our theory further corroborates the results in the study by Ihalainen-Talmander et al. [40] who found that MH stigma among nurses in PHC was related to the extent of clinical experience. Having a longer clinical experience made nurses feel more comfortable (i.e., a higher level of MHL) when encountering patients with MHPs. Relying on previous experience/clinical intuition was of great importance for the PHNs when being in the *“*professional comfort zone*”.* According to Welsh and Lyons [41], clinical intuition is crucial when it comes to assessing the needs of persons with MHP, which was evident in the present study. Clinical experience tended to determine the ability of nurses to extend the boundaries of clinical standards by using their intuition as well as their formal knowledge to meet the patients’ needs. Ever since the introduction of clinical intuition and its definition as having a rationale without an understanding [42], it has been criticized as a complex concept, and simply not a gut feeling [43]. The PHNs tended to describe their clinical intuition as “a simple gut feeling” not realizing or reflecting on all the experienced-based knowledge they had gathered during their clinical years. We would argue that the potential of PHNs as knowledge builders about MH needs to be acknowledged by the profession for them to be able to manage the encounters with confidence.

The collaborative care approach is one way of improving the management and the integration of MH in PHC [44–47], where developing the role of a care manager is one of the requirements. The care manager role concerns assisting and managing the patient by providing structured and systematic interventions [48]. In our result, being a care manager was related to the PHNs’ professional and autonomous role in PHC in MH encounters. Care management in PHC is described as an improvement in quality of care for patients with MHPs [48, 49]. The role of a care manager for MH in our findings was not experienced as being systematic and/or structured; rather it depended on the PHNs’ MHL. The subcategory *Calling for inter-professional learning* could thus be interpreted as a result of the lack of structured and systematic interventions within the PHC but also a description of the lack of implementation of the PHN role for MH in PHC. It was a question for the PHNs about referring the patient to the right person within or outside the organization. The lack of integrating MH in PHC created an uncertainty in the professional role [50]. The PHNs were frustrated about not having all the tools, support, or mandate to pursue and develop their clinical judgment and apply the standards of being a PHN to create actions that were in accordance with the PHNs moral and ethical standards. Björkman et al. [6] addressed the importance of building a trustful relationship for further encounters and found restriction of having insufficient MHL. The findings in this study suggested that a PHN who took the time and fully listened to the patient’s story lived up to the standards of being a PHN. A person-centered approach should be the baseline for all actions in the PHC context and not dependent on the persons’ reason for seeking care [23, 30]. PHNs as care managers need to be acknowledged by PHC and by policymakers in order to be able to improve mental health prevention and promotion from a holistic point of view [47].

Knowledge about MH was reported to have been improved over time, both within the profession and from an organizational perspective. However, in this study it was found that there are still gaps in the MH knowledge, as reflected by the subcategories targeting the lack of integration of MH in PHC and the need for inter-professional learning about encountering persons with MHP. The lack of integrating MH in a PHC context has left the PHNs being on their own to handle the encounter and this needs to be discussed from the perspective of patient rights and safety. It can be argued that if PHNs identify and approach MHP without building a relationship and not using routines and more standardized tools to verify their decision-making, persons with MHPs are at risk of not receiving adequate assessment and care. However, it is important to state that the PHNs in the present study most likely did the best they could when being on their own*.* They experienced that the organization failed to support them in their professional role. Furthermore, by not providing PHC with a holistic approach where MHP are considered equal to physical needs, also showed an insufficient support to the patients. There is a need to improve knowledge of MH in PHC [6, 8] and approach it in a systematic way in order to reduce MH stigma within the organization [40].

The clinical implications of our findings thus support a collaborative care approach [49]. The narratives of the PHNs helped to construct a theory and understand the conditions for recognizing, managing, and promoting MH in PHC, knowledge that could inform future intervention development to improve MHL. Further research is needed to understand patient perspectives, their previous experience, needs and preferences concerning PHC [51]. To involve both PHN and patients as stakeholders in the co-production of future PHC interventions is, however, vital for reducing MH inequalities.

## Limitations

In a constructivist grounded theory, the strengths of the preunderstandings of the authors are a contributing factor when conceptualization of the data [36]. This is also one of the discussed weaknesses against a theoretical sensitivity [52]. The authors’ background in this study could be seen as a strength of the theory sensitivity. First Author, novice in the practice of grounded theory but with experience as a PHN in different settings, primarily within PHC centers and School health services. Second author has background as occupational therapist within PHC centers, experience of grounded theory and a lecturer of the field. Third author is a PHN with experience of working in PHC centers, Municipality elderly care and associate professor in nursing, and forth a senior professor in MH with experience of grounded theory. Another study limitation was the difficulty in recruiting participants. It can be discussed whether it concerned the PHNs’ frustration, views, and attitudes when it comes to MH, their described uncertainty, and/or lack of knowledge about how to recognize, manage and promote MH that made participation challenging. Regarding non-responders, it would have been beneficial to understand why they declined participation, since a larger data set may have altered the results and affected the study’s resonance [36]. However, since it was a constructivist grounded theory approach, data guided the data collection process. The result should be viewed as a part of a larger puzzle and can be used to generate hypotheses for further research in the field.

## Conclusions

Being a relationship builder to initiate the dialogue was a complex construction depending on the PHNs’ MHL and the organizational capacity concerning MH. It cannot be assumed that all PHNs have the MHL needed to recognize MHP and to encounter patients with MHP, which thus creates a variety of ways to manage the encounters. The PHNs also experienced various degrees of organizational capacity about how to manage and support MH encounters, which led to frustration and feelings of being abandoned in the encounter. Narratives of the public health nurses helped to construct a theory and understand the conditions for recognizing, managing, and promoting mental health in primary health care, knowledge that could inform future intervention development and research to improve MHL.

## Supplementary Information

**Additional file 1.**

## Data Availability

The datasets generated and/or analysis during the current study are not publicly available due to ethical considerations from the participants’ point of view, but are available from the corresponding author on reasonable request.

## Abbreviations

LU: Lund University
MH: Mental health
MHL: Mental health literacy
MHP: Mental health problems
PHC: Primary health care
PHN: Public health nurse
